# Ocular vs. Cervical Vestibular Evoked Myogenic Potentials in Benign Paroxysmal Positional Vertigo: A Systematic Review and Meta-Analysis

**DOI:** 10.3389/fneur.2020.596454

**Published:** 2020-10-26

**Authors:** Gang Chen, Xiaoyan Dai, Xiuping Ren, Naifen Lin, Min Zhang, Zhaolin Du, Endong Zhang

**Affiliations:** ^1^Department of Otolaryngology Head and Neck Surgery, Shandong Provincial Hospital Affiliated to Shandong First Medical University, Jinan, China; ^2^Department of Anesthesiology, Shandong Provincial Hospital Affiliated to Shandong First Medical University, Jinan, China

**Keywords:** vestibular evoked myogenic potentials, benign paroxysmal positional vertigo, saccule, utricle, meta-analysis

## Abstract

**Objective:** To compare utricular dysfunction with saccular dysfunction in benign paroxysmal positional vertigo (BPPV), based on ocular vestibular evoked myogenic potentials (oVEMP) and cervical VEMP (cVEMP), respectively.

**Materials and Methods:** We performed a literature search exploring utricular and saccular dysfunction in BPPV patients through June 2020 using oVEMP and cVEMP, respectively. The databases included Pubmed, Embase, CENTRAL, CNKI, Wan Fang Data, and CBM. The literatures were limited to Chinese and English. Inclusion criteria and exclusion criteria were defined. We adopted abnormal rate as the outcome. All statistical processes were conducted through software Review Manager. Considering the air-conducted sound (ACS) and bone conducted vibration (BCV) may have different mechanisms, and three types of diagnostic criteria for abnormal VEMP were available, sub-group analysis was performed simultaneously according to the sound stimuli and the diagnostic criteria of abnormal VEMP.

**Results:** We retrieved 828 potentially relevant literatures, and finally 12 studies were included for meta-analysis of abnormal rate after duplication removal, titles and abstracts screening, and full-text reading. The abnormal rate of oVEMP was not significantly different from cVEMP (OR = 1.59, 95% CI = 0.99–2.57). But the abnormal rate was obviously different between the subgroups adopting ACS oVEMP and BCV oVEMP. In studies adopting ACS oVEMP, the abnormal rate of oVEMP was higher than cVEMP (OR = 1.85, 95% CI = 1.38–2.49). The abnormal rate of oVEMP was also higher than cVEMP when adopting asymmetry ratio (AR) and no response (NR) as diagnostic criteria (OR = 2.16, 95% CI = 1.61–2.89).

**Conclusion:** The meta-analysis reveals that utricular dysfunction may be more predominant in BPPV compared with saccular dysfunction.

## Introduction

Vestibular evoked myogenic potentials (VEMPs) have been widely adopted as a practical and effective measure of function of otolith pathway in central and peripheral vestibular disorders (1, 2). VEMPs can be recorded from the contracted sternocleidomastoid muscle (cervical VEMPs or cVEMPs) (3) and the inferior oblique muscle (ocular VEMPs or oVEMPs) (4). Generally, cVEMPs mainly represent the inhibitory vestibulo-collic reflex and reflect the functions of ipsilateral saccule and inferior vestibular nerve, while oVEMPs commonly represent the active vestibulo-ocular reflex and reflect predominantly the functions of contralateral utricle and superior vestibular nerve (5, 6).

VEMPs are short-latency alterations of myogenic activity in response to various stimuli. Loud air-conducted sound (ACS) (7) and bone conducted vibration (BCV) (8) are the most common stimulation modes adopted in clinical practice. The mechanisms of ACS and BCV may be different (9). In most cases, ACS is the best stimulus for cVEMP, while BCV oVEMP is better for detection of utricular dysfunction (5).

Benign paroxysmal positional vertigo (BPPV) is an episodic and brief vertigo or dizziness triggered by the sudden change of head position relative to gravity. BPPV is the most common cause of peripheral vertiginous disorders. So far the theories of canalolithiasis (10) and cupulolithiasis (11) have been widely regarded as the pathophysiology of BPPV. But the cause of otoconia detaching from macula of otolith organ remains unclear. In idiopathic BPPV, otolith dysfunction derived from degeneration of the utricular or saccular macula may be responsible for the dislodging of otoconia (12). Head trauma or inner ear diseases (13, 14) may especially damage the otolith organ, resulting in secondary BPPV. Due to the close anatomical relations, utricle is regarded as the principle source of otoconia debris and utricular dysfunction may be responsible for BPPV (15). However, a few of previous studies showed that otoconia may originate from the saccule and saccular dysfunction was correlated with BPPV occurrence and prognosis (16, 17).

There have been many studies which compared utricular function using oVEMP testing with saccular function using cVEMP testing in BPPV patients, and most studies confirmed that utricular dysfunction was more frequent (18, 19), but the conclusions were still contradictory (20, 21). Part of the reason may be different acoustic stimuli or different criteria for abnormal VEMP used by different studies. So we systemically retrieved all eligible studies and performed subgroup analysis simultaneously to compare the utricular and saccular dysfunctions in BPPV patients using oVEMP and cVEMP testing, respectively. The study aims to investigate whether utricular or saccular dysfunction may be predominant in BPPV.

## Materials and Methods

### Literature Search Strategy

**Figure 1:**
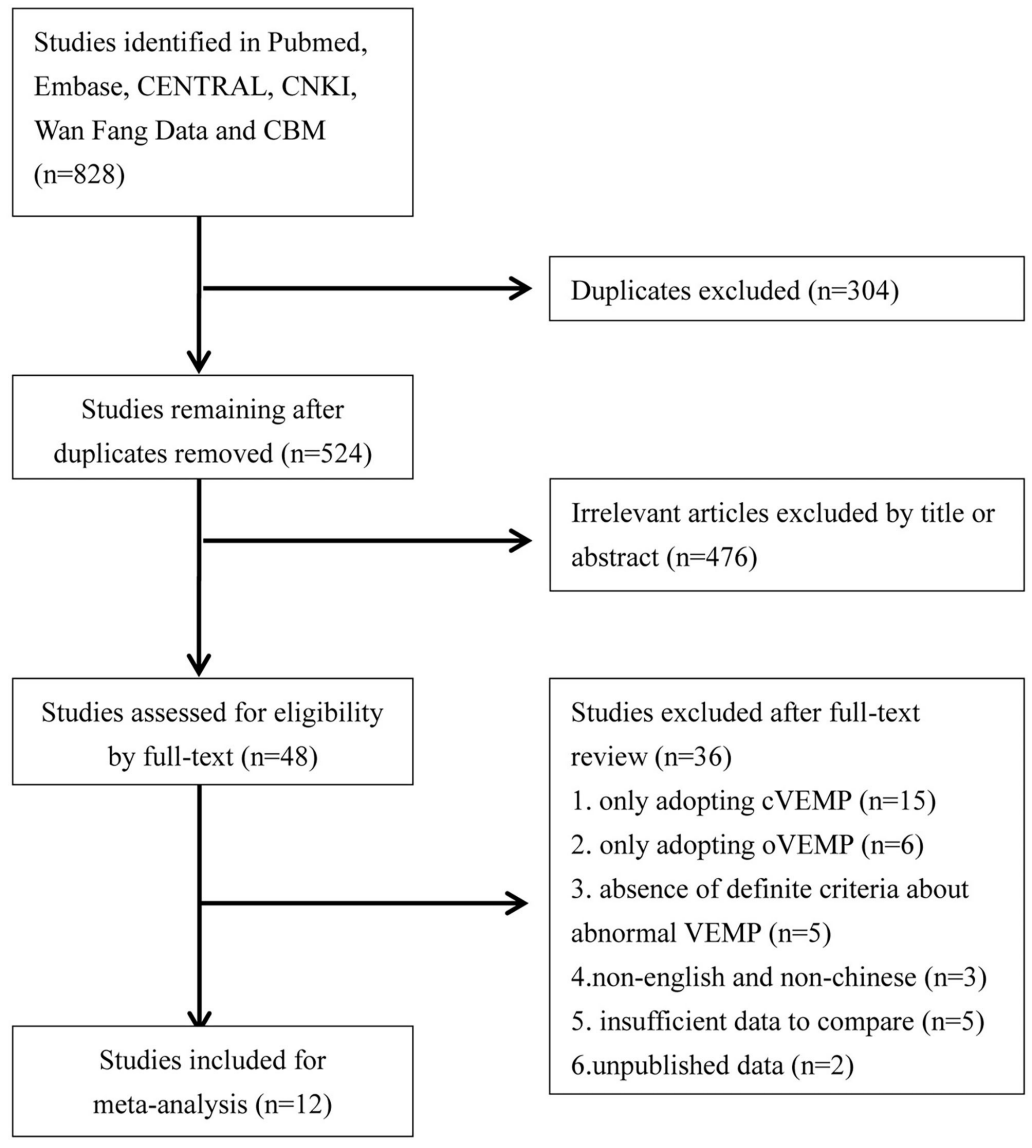
Flowchart of data search and studies selection for meta-analysis.

We performed a literature search which explored utricular and saccular dysfunction in BPPV patients through June 2020. The databases we systemically searched included Pubmed, Embase, CENTRAL, CNKI, Wan Fang Data, and CBM. The language was limited to Chinese and English. The search strategies were “vestibular evoked myogenic potential or VEMP” and “benign paroxysmal positional vertigo or BPPV.” We sequentially screened titles and abstracts, and then read full-text to identify literatures for meta-analyze. Additionally we screened all references of eligible literatures. The flowchart is presented in Figure 1.

### Study Selection Criteria

Inclusion criteria: (1) observation studies assessing utricular and saccular function in BPPV patients using oVEMP and cVEMP testing respectively; (2) diagnosis of BPPV relied on brief and recurrent vertigo and characteristic nystagmus in positional tests, such as Dix-Hallpike test and supine Roll test; (3) number of patients with response of oVEMP and cVEMP, or/and number of patients with abnormal oVEMP and cVEMP, were clearly stated.

Exclusion criteria: (1) insufficient data of oVEMP or cVEMP available resulting in incomparability; (2) absence of definite criteria for abnormal VEMP; (3) patents with conductive hearing loss, or other inner ear diseases, or neurological diseases; (4) unpublished studies, case reports, comments, practice guidelines, reviews, or letters.

### Outcome Synthesis

There has been no international consensus on diagnostic criteria for abnormal VEMP. Delayed peak latency might be attributed to the reducing nerve conduction velocity consequent on demyelination. Enlarged asymmetry ratio (AR) with VEMP response might indicate various degrees of damage involving the sensory organ of saccule and utricle, while absent VEMP response might mean the damage is extensive (21). Most relevant studies used abnormal rate of oVEMP and cVEMP to assess the functions of utricle and saccule. So in our meta-analysis, abnormal rate was adopted to compare utricular dysfunction with saccular dysfunction in BPPV patients.

### Data Extraction

Two authors (GC and XD) independently extracted all data through a uniform tool. Agreement was reached by consensus between the two authors. We extracted the data as follows: first author, country, publication year, age, gender, type of acoustic stimuli, criteria for abnormal VEMP, number of BPPV patients included, and number of patients with abnormal oVEMP and cVEMP.

### Statistical Analysis

All statistical processes of this systematic review were conducted using software Review Manager (RevMan), version 5.3. Dichotomous variables were analyzed by Odds ratios (OR) and its 95% confidence interval (CI). Statistical heterogeneity was evaluated by *X*^2^ and *I*^2^ index. The random-effects model was used if *I*^2^ > 50%, indicating significant heterogeneity, otherwise we chose fixed-effects model. Considering the ACS and BCV may have different mechanisms, the sub-group analysis according to acoustic stimulus was conducted. Besides, another sub-group analysis according to diagnostic criteria was conducted because three types of diagnostic criteria for abnormal VEMP were available.

## Results

### Literature Screening

We retrieved 828 potentially relevant literatures, and 304 literatures were removed for duplication, and 476 literatures were excluded for irrelevance to our purpose after screening titles and abstracts. Of the remaining 48 literatures needing a full-text reading, 15 were excluded for only adopting cVEMP, 6 were excluded for only adopting oVEMP, 3 were excluded for non-English and non-Chinese publication, 5 were excluded for absence of definite criteria for abnormal VEMP while comparing abnormal rate, 5 were excluded for insufficient data to compare through their studies, and 2 were excluded for unpublished data. Finally, we confirmed 12 studies for meta-analysis (18–29) (Figure 1).

### Characteristics of Studies Included

Of the 12 studies, 790 BPPV patients were involved, and 5 (25–29) were from China, and 4 (25, 27–29) were published in Chinese. All cVEMP testing in 12 studies and oVEMP testing in 11 studies were evoked by ACS, while oVEMP testing in 1 study (22) was evoked by BCV. Four studies (19, 21, 22, 24) adopted delayed latency and AR and no response (NR), and six studies (18, 23, 25, 27–29) adopted enlarged AR and NR, and two studies (20, 26) only adopted NR as their criteria for abnormal VEMP, respectively. The characteristics of included articles are described in Table 1.

**Table 1:** The basic characteristics of all eligible studies.

**References**	**Country**	**No. of BPPV**	**Gender (M:F)**	**Age (years) (mean ± SD)**	**Acoustic stimuli**		**Diagnostic critera of abnormal VEMP**	**No. with abnormal response**	
					**cVEMP**	**oVEMP**		**cVEMP**	**oVEMP**
Lee et al. (20)	Korea	36	NA	48.52 ± 10.06	ACS 90 dB nHL 500 Hz TB	ACS 95 dB nHL 500 Hz TB	NR	7	6
Nakahara et al. (19)	Japan	12	5:7	Mean 65.5	ACS 125 dB SPL 500 Hz TB	ACS 125 dB SPL 500 Hz TB	Latency + AR + NR	3	8
Talaat et al. (24)	Egypt	112	52:60	46.2 ± 10.2	ACS 95 dB nHL 500 Hz TB	ACS 95 dB nHL 500 Hz TB	Latency + AR + NR	15	11
Kim et al. (21)	Korea	102	48:54	62.8 ± 13.1	ACS 100 dB nHL 1,000 Hz TB	ACS 100 dB nHL 1,000 Hz TB	Latency + AR + NR	29	35
Zhou et al. (27)	China	59	13:46	48.35 ± 11.81	ACS 95 dB nHL 500 Hz TB	ACS 95 dB nHL 500 Hz TB	AR + NR	22	37
Xu et al. (26)	China	30	12:18	Mean 45.5	ACS 90 dB nHL 500 Hz TB	ACS 90 dB nHL 500 Hz TB	NR	9	17
Rao et al. (28)	China	40	7:33	51.36 ± 9.21	ACS 95 dB nHL 500 Hz TB	ACS 95 dB nHL 500 Hz TB	AR + NR	13	23
Fujimoto et al. (22)	Japan	99	32:67	63.0 ± 14.2	ACS 135 dB SPL 500 Hz TB	BCV 128 dB re 1 Mn 500 Hz TB	Latency + AR + NR	60	30
Tian et al. (25)	China	97	32:57^a^	NA	ACS 100 dB nHL 500 Hz TB	ACS 100 dB nHL 500 Hz TB	AR + NR	25	40
Martínez et al. (23)	Spain	67	16:51	Mean 58.06	ACS 100 dB 500 Hz TB	ACS 100 dB 500 Hz TB	AR + NR	33	41
Semmanaselvan et al. (18)	India	36	24:12	Mean 38.9	ACS 100 dB nHL 500 Hz TB	ACS 100 dB nHL 500 Hz TB	AR + NR	8	18
You et al. (29)	China	100	28:72	48.7 ± 5.8	ACS 100 dB nHL 500 Hz TB	ACS 100 dB nHL 500 Hz TB	AR + NR	44	30

*BPPV, benign paroxysmal positional vertigo; VEMP, vestibular evoked myogenic potentials; cVEMP, cervical VEMP; oVEMP, ocular VEMP; NA, not available; SD, standard deviation; No., number; AR, asymmetry ratio; NR, no response; ACS, air-conducted sound; BCV, bone-conducted vibration; TB, tone bursts*.

a *eight patients with bilateral absence of cVEMP or oVEMP waveform were not included*.

### Meta-Analysis Results

#### Abnormal Rate of cVEMP vs. oVEMP in BPPV Patients

**Figure 2:**
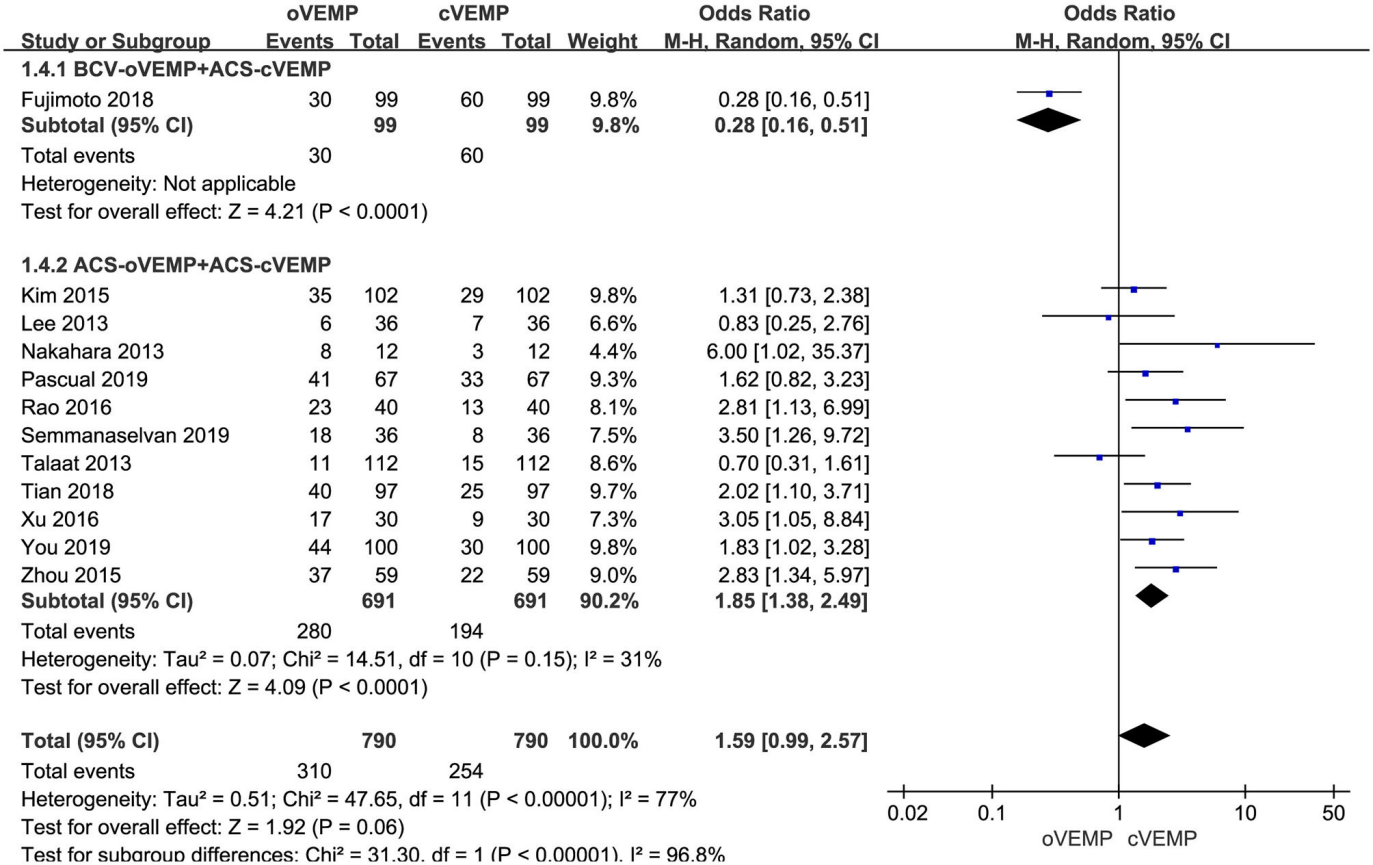
Meta-analysis of abnormal rate of vestibular evoked myogenic potential (VEMP) in benign paroxysmal positional vertigo (BPPV) patients based on sound stimuli.

Twelve studies assessed the abnormal rate of cVEMP vs. oVEMP in BPPV patients. Random-effects model was selected because of a significant heterogeneity (*p* < 0.00001, *I*^2^ = 77%, Figure 2). The abnormal rate of oVEMP in BPPV patients was not significantly different from cVEMP according to the forest plot (OR = 1.59, 95% CI = 0.99–2.57, *p* = 0.06, Figure 2).

In the sub-group analysis according to the sound stimuli, the result indicated a significant difference existed (*p* < 0.00001, *I*^2^ = 96.8%, Figure 2) between the one study adopting BCV oVEMP (OR = 0.28, 95% CI = 0.16–0.51, Figure 2) and eleven studies adopting ACS oVEMP (OR = 1.85, 95% CI = 1.38–2.49, Figure 2). In the subgroup adopting ACS oVEMP, the abnormal rate of oVEMP was significantly higher than cVEMP with mild heterogeneity (*p* < 0.0001, *I*^2^ = 31%).

**Figure 3:**
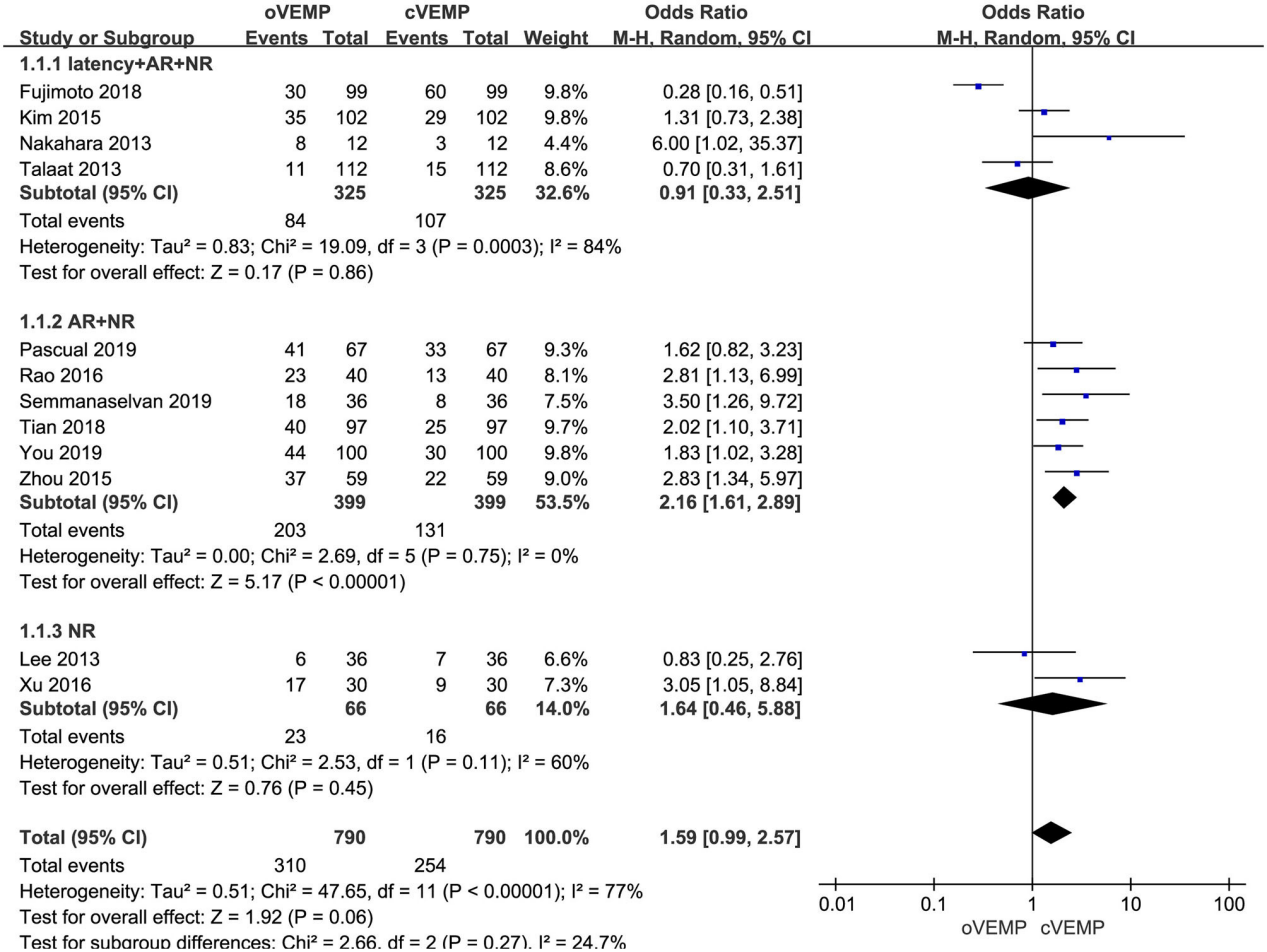
Meta-analysis of abnormal rate of vestibular evoked myogenic potential (VEMP) in benign paroxysmal positional vertigo (BPPV) patients based on diagnostic criteria of abnormal VEMP.

In the sub-group analysis according to the diagnostic criteria of abnormal VEMP, the result indicated no significant difference existed between the three groups (*p* = 0.27, *I*^2^ = 24.7%, Figure 3). In the first subgroup adopting delayed latency and enlarged AR and NR as diagnostic criteria (OR = 0.91, 95% CI = 0.33–2.51, *p* = 0.86, Figure 3), and the third subgroup adopting NR (OR = 1.64, 95% CI = 0.46–5.88, *p* = 0.45, Figure 3), the abnormal rate of oVEMP in BPPV patients was not significantly different from cVEMP. But six studies adopted enlarged AR and NR in the second subgroup (OR = 2.16, 95% CI = 1.61–2.89, *p* < 0.00001, Figure 3), and the abnormal rate of oVEMP in BPPV patients was significantly higher than cVEMP with no heterogeneity (*I*^2^ = 0%).

## Discussion

Several previous studies have compared oVEMP and cVEMP testing in BPPV patients. But the results varied widely. The abnormal rate of oVEMP ranged from 9.8% (24) to 66.7% (19), while cVEMP ranged from 13.4% (24) to 60.6% (22). The differences including the age of included individuals, stimulation mode, and diagnostic criteria for abnormal VEMP among each study may partly account for these.

Many studies (18, 19, 25) reported that the abnormal rate was higher compared with cVEMP in BPPV patients. From these studies, we may speculate that utricular dysfunction seems to be predominant in BPPV. But the argument is still under controversy. Semmanaselvan et al. (18) reported the opposite conclusion that the abnormal rate was lower compared with cVEMP. Talaat et al. (24) found that the proportion of abnormal cVEMP (13.4%) was higher than oVEMP (9.8%) although the difference was not statistically significant. Therefore, we conducted the meta-analysis and subgroup analysis to compare the abnormal rate of oVEMP with cVEMP in BPPV, and to investigate whether utricular or saccular dysfunction may be predominant in BPPV.

According to our meta-analysis, the difference of abnormal rate between cVEMP and oVEMP in BPPV patients was not significant, but the heterogeneity was very large (*I*^2^ = 77%). ACS and BCV are the most common acoustic stimuli modes adopted for cVEMP and oVEMP testing. In sub-group analysis according to the type of sound stimuli, the abnormal rate for oVEMP presented an expressive difference in the comparison between ACS and BCV. Only one included study (22) adopted BCV oVEMP, partly resulting in heterogeneity. Besides, the more important reasons for this huge difference may be that ACS and BCV have different stimulus translation mechanisms (9). Some otolith irregular neurons only respond to BCV, so BCV could evoke larger oVEMP responses (30). In detecting oVEMP abnormalities, ACS is more sensitive than BCV, while BCV shows a higher specificity (31). Therefore, we must be cautious about comparisons between ACS oVEMP and BCV oVEMP (9). In subgroups adopting ACS cVEMP and ACS oVEMP, the abnormal rate of oVEMP was higher than cVEMP with mild heterogeneity. This may indicate that utricular dysfunction may be more frequent in BPPV. Rosengren et al. (32) found the response rate of ACS cVEMP (96%) was higher than ACS oVEMP (81%) in normal subjects. The difference in the strength of ACS cVEMP and ACS oVEMP reflex pathways may account for this phenomenon. So we should consider this phenomenon in normal subjects or adopt normal controls in the further study about otolith dysfunction of BPPV patients.

There has been no international consensus on diagnostic criteria for abnormal VEMP, and the studies included in our meta-analysis adopted three types of diagnostic criteria. There was no difference about abnormal rate between them according to subgroup analysis, but the heterogeneity was large. Besides the large heterogeneity, few studies were included in the first subgroup adopting latency and AR and NR, and the third subgroup adopting NR, so we could not come to a convincing conclusion about the comparison of abnormal rate in the first and third subgroups. In the second subgroup adopting AR and NR as diagnostic criteria, six studies were included, and the abnormal rate of oVEMP was higher than cVEMP with no heterogeneity. This may also suggest that utricular dysfunction may be more common in BPPV, and the studies have comparability if adopting AR and NR as diagnostic criteria. In cVEMP testing of BPPV patients, latency of p13 was prolonged regardless of the age (33). But the latency parameter of VEMP waveform is particularly affected by rise time and stimulus shape (5). Two studies used latency criteria from their own normal controls, while two studies adopted latency criteria from other researchers. These may add the heterogeneity when including latency as diagnostic criteria. We should verify the reliability of using delayed latency as diagnostic criteria in future studies with large sample and uniform parameters of VEMP testing.

A few limitations still remain be considered in our study. First of all, a part of the studies adopted different stimulation modes, such as ACS and BCV. Even if they all adopted ACS, the intensity and frequency of acoustic stimuli may have a little difference. And only one study on BCV oVEMP was included in our meta-analysis. Secondly, the different diagnostic criteria for abnormal VEMP resulted in large heterogeneity. Thirdly, the mean ages of BPPV individuals in the included articles were different from each other, and normal control group was absent. Therefore, we should conduct well-designed studies with large sample and normal control group and uniform parameters of VEMP testing to further investigate the otolith dysfunction of BPPV patients.

## Conclusion

In oVEMP, the abnormal rate has been higher using ACS when compared to BCV, showing that BCV seems to be more specific for the evaluation of utricular dysfunction. And in studies adopting ACS cVEMP and ACS oVEMP, the abnormal rate of oVEMP was higher than cVEMP. And the abnormal rate of oVEMP in BPPV patients was also higher than cVEMP with no heterogeneity if adopting AR and NR as diagnostic criteria. It is inferred that utricular dysfunction may be more predominant in BPPV compared with saccular dysfunction. Well-designed studies with large sample and normal control group and uniform parameters of VEMP testing should be conducted to further investigate the otolith dysfunction of BPPV patients.

## Data Availability Statement

The original contributions presented in the study are included in the article/supplementary materials, further inquiries can be directed to the corresponding author/s.

## Author Contributions

GC and XD contributed to the study design, statistical analysis, and manuscript draft. All authors helped to perform the analysis and to revise the manuscript with constructive discussions.

## Conflict of Interest

The authors declare that the research was conducted in the absence of any commercial or financial relationships that could be construed as a potential conflict of interest.
